# Editorial: Probiotics and bioactive agents in modulating harmful oral biofilms

**DOI:** 10.3389/fmicb.2026.1877514

**Published:** 2026-06-19

**Authors:** Leon G. Leanse, Alessandra Nara de Souza Rastelli, Asad U. Khan, George Grant

**Affiliations:** 1 Health and Sport Sciences Hub, University of Gibraltar, Gibraltar, Gibraltar; 2 Faculty of Dentistry, São Paulo State University-UNESP, Araraquara, Brazil; 3 Antimicrobial Resistance Laboratory, Interdisciplinary Biotechnology Unit, Aligarh Muslim University, Aligarh, India; 4 Independent Researcher, Aberdeen, United Kingdom

**Keywords:** commensal microbes, neurological dysfunction, novel treatments, oral ailments, oral biofilms, pathobionts, systemic diseases

The oral microbiome comprises over 700 species of bacteria, fungi, viruses, and archaea associated with the mouth and tongue epithelia, dental plaque, and saliva. Its composition varies by site, but bacteria are predominant, primarily of the phyla *Actinomycetota, Bacteroidota, Bacillota, Fusobacteriota, Pseudomonadota, Saccharibacteria*, and *Spirochaetota*, with *Streptococcus, Prevotella, Veillonella, Lactobacillus, Actinomyces*, and *Neisseria* as dominant genera. Most members are commensals that support oral health and immune homeostasis, whereas pathogenic species, including *Porphyromonas gingivalis, Streptococcus mutans, Tannerella forsythia*, and *Fusobacterium nucleatum*, typically persist at low abundance but can proliferate when oral homeostasis is disrupted by dietary, environmental, or systemic factors (Baker et al., 2024; Rajasekaran et al., 2024; Tian et al., 2024; Morrison et al., 2023).

Oral microbes persist in the hostile oral environment primarily through the formation of surface-associated biofilms. These structured microbial communities are embedded in an extracellular polymeric matrix that confers protection against host immunity, antimicrobial agents, and environmental factors. As reviewed by Lv et al. healthy oral biofilms are dominated by commensal bacteria that support epithelial integrity, regulate local immune responses, and inhibit pathogen colonization. However, disruptive oral conditions can shift biofilm composition toward pathogenic species. Within the biofilm, these organisms exhibit enhanced resistance to immune clearance and antimicrobial treatment, contributing to persistent disease (Almatroudi, 2025; Shalshabilla and Hermawan, 2025; Baker et al., 2024; Ray, 2024a,b). As a result, emerging therapeutic strategies increasingly focus on modulating biofilm composition rather than complete biofilm eradication (Thamir and Hasan, 2026; Wu et al., 2026).

## Oral disorders

Oral biofilms harbor pathogenic bacteria, viruses, and mycobionts that contribute to major oral diseases, including dental caries, periodontitis, and oral cancers. Increasing evidence, particularly in oral oncology, indicates that disease outcomes are shaped not only by pathogenic members but also by commensal microbes that can curb or out compete deleterious species. Accordingly, alongside antimicrobial strategies aimed at disrupting pathogenic biofilms, approaches that selectively enhance beneficial microbial populations may represent effective complementary interventions for the management of biofilm-associated oral diseases (Di Pierro et al.; Lv et al.; Łasica et al.; Pérez-Losada et al.; Popowski et al.; Glavina et al., 2026; Chiscuzzu et al., 2025; Foroughi et al., 2025; Govindarajan et al., 2025; Priya et al., 2023; Baker et al., 2024).

Łasica et al. provide a comprehensive review of the inflammatory etiology of periodontitis that results in progressive damage to gingival tissues, periodontal ligaments, cementum, and alveolar bone, ultimately leading to tooth loosening and loss. Beyond oral pathology, periodontitis is associated with systemic, cerebral, and reproductive disorders. Conventional management approaches, including oral hygiene improvement, mechanical plaque removal, and antimicrobial therapy, remain effective in reducing pathogen burden; however, rising antibiotic resistance [microbes within biofilms may be up to 1,000 times more resistant to antibiotics than their free counterparts] and the failure of standard techniques to fully eliminate the deleterious microbes substantially limit their long-term efficacy. Alternative antimicrobial strategies, such as bacteriophage therapy and bacteriotherapy, have therefore gained attention. Bacteriophages can selectively inhibit oral pathogens, but their clinical application remains constrained by limited phage availability. Predatory Gram-negative bacteria, including *Bdellovibrio* and related organisms (Salah et al., 2025), have demonstrated the ability to reduce key periodontal pathogens (*Aggregatibacter actinomycetemcomitans* and *F. nucleatum*) in biofilm models, promote beneficial microbial communities, and exhibit protective effects in pre-clinical animal models. Further research is required to elucidate their mechanisms of action and clinical potential.

Oral infections extend beyond epithelial and dental surfaces and represent a major challenge in oral and maxillofacial surgery. Popowski et al. and Choi and Hong (2026) report that pathogen-containing biofilms associated with local tissues and prosthetic materials, which are often highly susceptible to bacterial colonization, contribute to adverse clinical outcomes, including postoperative infections, delayed healing, impaired prosthetic integration, and surgical failure. Autogenous bone remains the preferred grafting material, but its limited availability has increased reliance on xenogeneic and alloplastic substitutes, which are susceptible to microbial colonization and require careful control. Emerging antimicrobial biomaterials, including silver- and strontium-doped hydroxyapatite, copper- and zinc-enriched bioglasses, and chitosan-based calcium phosphate scaffolds, show promise by coupling osteogenic potential with biofilm suppression. Nevertheless, strict infection control measures, such as preoperative antiseptic rinses, single-dose antibiotic prophylaxis, and meticulous surgical techniques, remain critical for minimizing microbial risk and ensuring clinical success.

## Systemic and intestinal disorders

Oral biofilm pathogens, including *P. gingivalis* and *F. nucleatum*, are increasingly linked to the risk, incidence, and severity of multiple systemic conditions. These include respiratory diseases, type 2 diabetes mellitus, cardiovascular disease, rheumatoid arthritis, bone disorders, hepatic and metabolic dysfunctions, inflammatory bowel diseases, and both intestinal and systemic cancers. These disorders may arise directly via microbial translocation to distal tissues, or indirectly through the systemic dissemination of virulence factors and microbial metabolites. In addition, oral microbes or their metabolic products may reshape the gastrointestinal microbiome and metabolome (Łasica et al.; Pérez-Losada et al.; Glavina et al., 2026; Bruno et al., 2025; Harrandah, 2025; Hu et al., 2025; Kumar and Tyagi, 2025; Kurtzman et al., 2025a,b; Nayak et al., 2025; Zhang and Zhang, 2025; Baker et al., 2024).

Pérez-Losada et al. demonstrated that oral dysbiosis is associated with allergic rhinitis (AR) and its progression to asthma (ARAS). Patients with AR or ARAS showed increased abundance of *Fusobacteriota* and *Pseudomonadota*, along with reduced abundance of *Actinobacteriota* and *Bacillota*. At the genus level, *Fusobacterium, Gemella, Haemophilus, Leptotrichia, Neisseria, Porphyromonas*, and *Prevotella* were enriched, whereas *Actinomyces, Streptococcus*, and *Veillonella* were depleted. Functional analyses indicated upregulation of metabolic pathways linked to allergic sensitization, airway inflammation, and immune responses, supporting oral dysbiosis as a contributory factor in AR onset and progression to ARAS.

*Akkermansia muciniphila*, traditionally recognized as an intestinal commensal, is also detectable in saliva and has been shown to suppress oral inflammation and inhibit periodontal pathogens, including *P. gingivalis* and *F. nucleatum* (Anderson et al., 2024). Recent *in vivo* research by Zhang et al. demonstrates that patients with severe periodontitis exhibit marked gut dysbiosis, characterized by elevated pathogenic taxa and reduced levels of beneficial microbes, such as *A. muciniphila*. The abundance of *A. muciniphila* was partially restored in patients following conventional periodontal therapy. In a murine model of periodontitis, which displayed inflammation and compromised intestinal barrier integrity, fecal levels of *A. muciniphila* were also greatly reduced. Transplantation of the fecal microbiota from periodontitis patients to these mice perpetuated both alveolar bone loss and intestinal pathology, whereas transplantation from healthy donors substantially ameliorated deleterious effects. Together, these findings underscore the bidirectional oral–gut axis, whereby gut microbial composition and metabolites influence the severity of periodontitis, while oral inflammatory states reciprocally modulate gut microbiome structure and intestinal health.

## Brain dysfunction

Growing evidence links oral biofilm-associated pathogens with an increased risk and severity of neurological and neuropsychiatric conditions, including Alzheimer's disease, Parkinson's disease, autism spectrum disorder, anxiety, depression, and age-related cognitive decline. Although the underlying mechanisms remain poorly defined, these effects may involve the translocation of oral microbes or their metabolites via an oral–brain axis, or indirect signaling through the oral–gut–brain axis (Łasica et al.; Glavina et al., 2026; Adil et al., 2025; Borrego-Ruiz and Borrego, 2025; Clasen et al., 2025; Kong et al., 2025; Kumar and Tyagi, 2025; Miranda et al., 2026; Rozenblum et al., 2025; Baker et al., 2024).

## Treatments

### Biofilm modulation

Multiple agents and techniques are used to disrupt oral biofilms and suppress periodontal pathogens. However, while generally effective, they are often non-selective, eliminating both commensal and pathogenic microbes (Fydrych et al., 2025; Narwal et al., 2025). Studies to find more discriminating treatment procedures are of high importance (Ciren et al., 2026; Wu et al., 2026). Bueno-Silva et al. evaluated an oral gel containing *Aloe vera*, propolis extract, green tea, cranberry, and calendula on a multispecies oral biofilm model comprising 33 bacterial species. When applied as a toothpaste alternative (eight 1-min applications at 12-h intervals), the formulation significantly reduced total bacterial counts, although its efficacy was lower than that of chlorhexidine + cetylpyridinium chloride or stannous-fluoride toothpastes. Notably, a single 12-h treatment produced marked effects, reducing biofilm metabolic activity by 45% and total bacterial load by 59% across 24 species. Substantial reductions were observed in *F. nucleatum polymorphum, Prevotella intermedia*, and *P. gingivalis*, with a particularly pronounced decrease in *T. forsythia*, a key pathogen in periodontitis and peri-implantitis. Siddiqui et al. studied a new herbal mouthwash (*Azadirachta indica, Calendula officinalis, Echinacea purpurea*, and *Plantago major*) *in vitro* and *ex vivo*. The formulation selectively inhibited key periodontal pathogens (including *F. nucleatum* and *P. gingivalis*) while sparing commensals (e.g., *Streptococcus oralis, Streptococcus gordonii*, and *Veillonella parvula*). In a mixed-species biofilm model, pathogen burden fell markedly without disrupting overall biofilm integrity. In studies with pure compounds, Dong et al. evaluated whether the human cathelicidin-derived antimicrobial peptide LL-37 and polymyxin B could dampen LPS-driven inflammation in saliva and subgingival biofilm samples from healthy, gingivitis, and periodontitis patients. Pretreatment with either peptide reduced THP-1 monocyte inflammatory responses, attenuating NF-κB/IRF signaling and lowering IL-1β, IL-6, IL-8, and TNF-α, with minimal effects on anti-inflammatory mediators (TGF-β, IL-10). Taudte et al. assessed S-0636, a small-molecule inhibitor of glutaminyl cyclase (present in *P. gingivalis, T. forsythia*, and *P. intermedia*). When *P. gingivalis* was used as a test model, S-0636 strongly inhibited the enzyme and reduced bacterial virulence by >70% at ≤ 62.5 μM. By contrast, it had no detectable effects on oral commensals at 0.25 mM. Furthermore, Eick et al. showed that S-0636 suppressed a 12-species oral biofilm model: at ≥125 μM, it reduced biomass, metabolic activity, and virulence. Monocytes exposed to bacteria from treated biofilms secreted much less IL-1β, while IL-10 was unchanged. Overall, these studies highlight several possible treatments for managing subgingival and periodontal biofilms that merit *in vivo* testing and validation.

### Biotics

Probiotics, prebiotics, synbiotics, and postbiotics have been evaluated as adjuncts for oral dysbiosis, but clinical responses remain variable (Ghamari et al., 2026). In a comprehensive review, Di Pierro et al. summarize evidence from *in vitro* studies, animal models, and clinical trials, with particular focus on *Streptococcus salivarius* K12 and M18. These strains may support Eubiosis and suppress oral pathogens; however, *in vivo* efficacy is inconsistent. The authors propose chlorhexidine pre-treatment prior to probiotic administration to reduce competing taxa and facilitate probiotic colonization. The recent review by Anderson et al. (2024) and the experimental studies by Zhang et al. highlight the potential of *A. muciniphila* as a probiotic or live biotherapeutic product (LBP) for managing oral microbe-linked disorders, including periodontitis. As an alternative to live probiotics against oral pathogens, Zanetta et al. investigated cell-free supernatants (CFS) from *Limosilactobacillus reuteri* LRE11, *Lacticaseibacillus rhamnosus* LR04, and *Lacticaseibacillus casei* LC04 (single strains or mixed culture) on FaDu hypopharyngeal squamous carcinoma cells infected with *A. actinomycetemcomitans, F. nucleatum*, and *P. gingivalis*. CFS reduced IL-6 production and infection-associated cytotoxicity; proteomics identified metabolic enzymes, ribosomal/transport proteins, and defense proteins, alongside potentially anti-inflammatory short-chain fatty acids. Vitamin D further enhanced protection, supporting probiotic-derived (viable, heat-killed, or CFS) strategies, with or without vitamin D, for managing oral disease and potentially modulating the tumor microenvironment. Scheithauer et al. assessed *Saccharomyces cerevisiae*, yeast extracts, and yeast cell wall preparations in oral biofilm models derived from periodontitis-positive and healthy donors. Yeast cell wall preparations reduced biofilm density and dysbiosis *in vitro*, lowered key pathogens (e.g., *T. forsythia*), and increased health-associated taxa. They also reduced short-chain fatty acid production (acetic and butyric acids, among others). Whole yeast and extracts produced qualitatively similar but weaker effects. The mechanism remains unclear but may relate to polysaccharide-rich cell wall components (β-glucans and mannans) that disrupt biofilm structure and/or impede pathogen adhesion, thereby promoting Eubiosis and a healthier metabolic profile. Collectively, these findings support yeast cell wall products as candidate adjuncts for preventing or managing periodontitis and other oral biofilm-associated disorders.

### Antimicrobial photodynamic therapy

In an alternative approach, Li et al. reviewed antimicrobial photodynamic therapy (aPDT), a non-invasive treatment combining a photosensitizer, visible light, and oxygen to generate reactive oxygen species that disrupt biofilms and kill microbial cells. This process is rapid, tissue-sparing, and active against antibiotic-resistant strains. Although it may impact commensals within oral biofilms, optimized photosensitizers and protocols can preferentially target pathogens. Overall, aPDT shows promise as an adjuvant for the management of oral biofilm-associated diseases.

### Summary

Oral biofilms in health are largely commensal and support epithelial integrity and immune homeostasis. When ecological balance is disrupted, pathogen-enriched biofilms drive oral disease and can contribute to intestinal, systemic, and neuroinflammatory disorders via internal dissemination of pathogenic microbiota, virulence factors, and metabolites. While conventional approaches can reduce harmful oral biofilms, emerging strategies aim to selectively suppress pathobionts while preserving biofilm structure and promoting beneficial commensal microbiota. New strategies that could support this approach are outlined here.
